# Trends in Epidemiology of COPD in HIV-Infected Patients in Spain (1997–2012)

**DOI:** 10.1371/journal.pone.0166421

**Published:** 2016-11-15

**Authors:** Javier de Miguel-Díez, Ana López-de-Andrés, Rodrigo Jiménez-García, Luis Puente-Maestu, Isabel Jiménez-Trujillo, Valentín Hernández-Barrera, Salvador Resino, Alejandro Álvaro-Meca

**Affiliations:** 1 Pneumology Department, Hospital General Universitario Gregorio Marañón, Facultad de Medicina, Universidad Complutense de Madrid, Instituto de Investigación Sanitaria Gregorio Marañón (IiSGM), Madrid, Spain; 2 Preventive Medicine and Public Health Teaching and Research Unit, Department of Health Sciences, Universidad Rey Juan Carlos, Alcorcón, Madrid, Spain; 3 National Centre of Microbiology, Instituto de Salud Carlos III, Majadahonda, Madrid, Spain; Central University of Tamil Nadu, INDIA

## Abstract

**Purpose:**

The aim of this study was to estimate trends of incidence of hospital admissions and in-hospital mortality (IHM) in HIV-infected patients with COPD in the combination antiretroviral therapy (cART) era in Spain (1997–2012).

**Methods:**

A retrospective study with data from nationwide population-based COPD diagnoses in the Spanish Minimum Basic Data Set (MBDS) was performed. We established groups according to their HIV and HCV infections: 1) HIV-uninfected patients; 2) HIV-infected patients (with or without HCV coinfection).

**Results:**

1,580,207 patients discharge with a COPD diagnosis were included in the study, 8902 of them were HIV-infected patients (5000 HIV-monoinfected patients and 3902 HIV/HCV-coinfected patients). The HIV-infected patients had higher incidence rates of hospital admissions for COPD than the HIV-uninfected patients during the study period. The HIV-monoinfected patients had higher rates of hospitalizations for COPD than the HIV/HCV-coinfected patients in the early-period cART (1997–1999), but these rates decreased in the first group and increased in the second, being even similar in both groups in the late-period cART (2004–2011). On the other hand, the HIV-infected patients with COPD had higher IHM than the HIV-uninfected patients with COPD. The mortality rates were higher in the HIV-monoinfected patients with COPD than in the HIV/HCV-coinfected patients with COPD in the early-period cART; however, in the late-period cART, the mortality rates trends seems higher in the HIV/HCV group. The likelihood of death in HIV/HCV-coinfected patients with COPD was similar to than in HIV-monoinfected patients with COPD.

**Conclusions:**

Incidence of hospital admissions for COPD and IHM have decreased among HIV-monoinfected individuals but have increased steadily among HIV/HCV-coinfected individuals in the cART era.

## Introduction

The introduction of combination antiretroviral therapy (cART) has resulted in a decline in infectious complications and mortality in persons living with the human immunodeficiency virus (HIV)[1,2]. With the increases in life expectancy, the incidence of nonopportunistic lung diseases, such us chronic obstructive pulmonary disease (COPD) has become more common in these individuals. Indeed, recent studies have shown an increased prevalence of COPD in HIV-infected patients in comparison with their HIV-uninfected counterparts [3,4].

Although the pathogenic mechanisms of HIV-associated COPD remains unclear [5], it may be due in part to higher prevalence of smoking in these individuals [6]. Other risk behaviors, such as use of injected and inhalational drugs, also damage the lungs [7]. In addition, pulmonary infections such as bacterial pneumonia and pulmonary colonization by *Pneumocystis jirovecii* may contribute to the pathogenesis of COPD [8,9]. Irrespective of these risk factors, HIV infection is being considered as an independent risks factor for COPD [3,10]. Cohort studies have also found an association between antiretroviral therapy and COPD, although the mechanism of this relationship is not well understood. Potential explanations include direct effects of these drugs, restoration of the immune system allowing for an increased inflammatory response after therapy is initiated, or the development of autoimmunity [5].

The studies conducted to date about HIV-related COPD have included specific populations with or without history of smoking or intravenous drug use and African-American or Hispanic minorities in the USA, but few studies have been performed in Europe [11–14]. On the other hand, although some studies have examined the trends in causes of death among persons diagnosed with HIV-infection [15,16], there are virtually no specific studies about trends in incidence and mortality of COPD in HIV-infected individuals in the cART.

Hepatitis C virus (HCV) infection has also been implicated as a potential viral mediator of obstructive lung disease development [17]. It is recognized to trigger a chronic inflammatory response. Several epidemiological studies have suggested that HCV could be a risk factor for COPD [18–20]. However, recent findings have suggested that HCV may not be a sole contributor to the increased prevalence of COPD [21]. So, the effect of HCV infection could be magnified in HIV/HCV coinfected patients [22].

The aim of this study was to estimate trends of incidence of hospital admissions and in-hospital mortality (IHM) for COPD in HIV-infected patients in the cART era in Spain, with particular attention to HIV/HCV-coinfected patients.

## Material and Methods

### Study period and population

A retrospective study with data from nationwide population-based COPD diagnoses in the Spanish Minimum Basic Data Set (MBDS) was performed. Among patients with a COPD diagnosis we identified HIV-infected subjects over 15 years in age in Spanish hospitals from 1 January 1997 to 31 December 2012. We subdivided the study period into three calendar periods when different cART regimens were available in Spain [23,24]: from 1997 to 1999 (early-cART period), from 2000 to 2003 (mid-cART period), and from 2004 to 2012 (late-cART period).

Data were obtained from the records of the MBDS of the National Surveillance System for Hospital Data in Spain, provided by the Spanish Ministry of Health. The MBDS is a clinical and administrative database containing information obtained and recorded at time of hospital discharge, with an estimated coverage of 97.7% of total hospital admissions to public hospitals [25]. The MBDS provides the encrypted patient identification number, sex, date of birth, dates of hospital admission and discharge, medical institutions providing the services, the diagnosis and procedure codes according to the *International Classification of Diseases*, *9th ed*, *Clinical Modification* (ICD-9-CM), and outcome at discharge [26].

Data confidentiality was maintained at all times in accordance with Spanish legislation. Patient identifiers were deleted by the Spanish Ministry of Health before the database was provided to the authors in order to maintain patient anonymity. It is not possible to identify patients individually, either in this article or in the database. Since the dataset was anonymous and mandatory, informed consent was unnecessary. The Spanish Ministry of Health considered that our study protocol fulfilled all ethical requirements according to Spanish legislation and provided us with the database. Given the nature of the investigation and according to the Spanish Legislation approval of an Ethics Committee is not required. Anonymized data was used and authors were not involved with the patients' medical treatment nor had any interaction with the participants and none of the authors were affiliated with the hospitals/clinics where patients were treated.

### ICD-9-CM codes and study groups

We selected all patients who were coded in the MBDS with a diagnosis of COPD (ICD-9 codes 490, 491, 492, 494, or 496) in any diagnosis position. The ICD-9-CM codes were also used for defining the viral infection status: 1) chronic HIV infection (ICD-9-CM codes 042 or V08); 2) chronic HCV infection (ICD-9-CM codes 070.44, 070.54, 070.7x, or V02.62); 3) chronic HBV infection (ICD-9-CM codes 070.2x, 070.3x, or V02.61). Next, we established several groups of patients according to their HIV and HCV infections: 1) HIV-uninfected patients (patients without HIV or HCV infections); 2) HIV-infected patients (HIV-infected patients with or without HCV coinfection). This last group was divided into two groups according to their HCV infection: a) HIV-monoinfected patients (patients solely infected with HIV); b) HIV/HCV coinfected patients (patients coinfected with HIV and HCV). HBV infection was a criterion for exclusion. Besides, hospitalizations due to acute hepatitis C (ICD-9-CM codes 070.41 and 070.51) were also ruled out because our objective was to evaluate the epidemiology of COPD in patients with HIV and HCV chronic infections.

### Outcome variables

The index episode was defined as the occurrence of a hospital discharge with diagnosis of COPD and allied conditions according to the ICD-9-CM codes (490 to 496). Patients who were readmitted with a COPD diagnosis were not counted as new episodes of COPD. The outcome variables analyzed were the following: 1) new COPD diagnosis (incidence); 2) in hospital mortality among patients with a COPD diagnosis (intrahospital COPD mortality).

### Estimation of the number of people living with HIV/AIDS in Spain

To assess incidences we needed an estimation of the number of people living with HIV/AIDS in Spain. This estimation was provided by the National Centre of Epidemiology (*Instituto de Salud Carlos III*, *Madrid*, Spain) [27]. This estimation was done using the Estimation and Projection Package (EPP) and Spectrum software, two programs utilized by the Joint UNAIDS/WHO for estimating and projecting HIV prevalence levels in countries with concentrated epidemics [28,29].

### Estimation of the number of individuals coinfected with HIV and HCV in Spain

The number of subjects older than 15 coinfected with HIV and HCV in Spain was estimated using surrogate data. For this purpose, we reviewed the results from the hospital survey of HIV/AIDS infected patients, a second-generation surveillance system in people living with HIV coordinated by the National Centre of Epidemiology [30], and the reports of two Spanish national cohorts: the “Grupo de Estudio de Sida” (GeSIDA) [31] and the “Asociación Médica VACH de Estudios Multicentricos (AMVACH)” [32]. From these sources we obtained the percentage of patients with HCV antibodies. The number of subjects coinfected with HIV/HCV was the result of multiplying the number of individuals infected with HIV and the percentage of patients with HCV antibodies. With this data, we made a regression model which provided us the number of subjects coinfected with HIV and HCV in Spain.

### Statistical analysis

A retrospective design was used to evaluate the trends of COPD. We estimated the rates incidence and intrahospital mortality among those patients with COPD diagnoses. The number of events within each calendar year or calendar period was used as numerator. The denominator was the number of persons at risk within each calendar year or calendar period. For the HIV-uninfected patients, we used the number of persons censored in Spain (National Statistics Institute; http://www.ine.es/); for the HIV-infected patients, we used the estimation of the number of subjects living with HIV/AIDS in Spain; for HIV/HCV-group HIV/HCV-coinfected patients, we used the estimation of the number of subjects coinfected with HIV and HCV in Spain; and for the HIV-monoinfected patients we used the difference between number of subjects infected with HIV and number of subjects coinfected with HIV and HCV.

Overall, results are presented as the mean (95% confidence interval (95% CI)) for continuous variables, frequencies and percentages for categorical data. Categorical data and proportions were analyzed using chi-squared test or Fisher’s exact test, as required. T-Test or Mann-Whitney U test were used to compare continuous variables. The incidence and mortality were compared using Poisson distribution. Temporal trends of incidence and mortality rates of COPD were evaluated using Poisson distribution. We also calculated the odds for in-hospital death in patients with a COPD diagnosis according to HIV and HCV status using logistic regression models, which were adjusted by age, sex, and Charlson co-morbidity index (CCI) [33]. Tobacco abuse (ICD-9-CM codes 305.1 or V15.82) was also included in the regression model.

Statistical analysis was performed using the R statistical package version 3.2.2 (GNU General Public License) [34]. All tests were two-tailed with p-values <0.05 considered significant. Bonferroni penalization was used to adjust statistical significances for multiple comparisons.

## Results

### Study population

Table 1 shows the clinical and epidemiological characteristics of the 1,580,207 patients discharge with a COPD diagnosis included in the study, 8902 of them were HIV-infected patients (5000 HIV-monoinfected patients and 3902 HIV/HCV-coinfected patients).

**Table 1:** Epidemiological and clinical characteristics of patients with a hospital admission and a COPD diagnosis from 1997 to 2012 in Spain.

	1a: HIV-uninfected versus HIV-infected patients			1b: HIV-monoinfected vs HIV/HCV coinfected patients		
	Non-HIV	All HIV	p-value	HIV group	HIV/HCV group	p-value
**No. of patients**	1571305	8902		5000	3902	
**Males**	1216676 (77.4)	6569 (73.8)	**<0.001**	3705 (74.1)	2864 (73.4)	0.470
**Age (years)**	71.32 (71.3; 71.34)	44.72 (44.49; 44.94)	**<0.001**	45.82 (45.47; 46.17)	43.31 (43.07; 43.55)	**<0.001**
**Length of stay (days)**	10.23 (10.21; 10.25)	11.8 (11.51; 12.08)	**<0.001**	12.51 (12.09; 12.93)	10.88 (10.51; 11.24)	**<0.001**
**Charlson index**	2.43 (2.43; 2.43)	1.5 (1.47; 1.53)	**<0.001**	1.57 (1.53; 1.61)	1.41 (1.37; 1.45)	**<0.001**
**In-hospital death**	108440 (6.9)	416 (4.7)	**<0.001**	258 (5.2)	158 (4)	**0.016**
**Substances of abuse**						
Drugs	314135 (20)	5791 (65.1)	**<0.001**	2796 (55.9)	2995 (76.8)	**<0.001**
Alcohol	45146 (2.9)	434 (4.9)	**<0.001**	184 (3.7)	250 (6.4)	**<0.001**
Tobacco	494045 (31.4)	4201 (47.2)	**<0.001**	2118 (42.4)	2083 (53.4)	**<0.001**
**Conditions influencing in health status**						
Surgical conditions	137934 (8.8)	265 (3)	**<0.001**	166 (3.3)	99 (2.5)	**0.036**
Trauma	38575 (2.5)	188 (2.1)	**0.041**	93 (1.9)	95 (2.4)	0.072
**Comorbid diseases**						
Myocardial Infarction	92765 (5.9)	122 (1.4)	**<0.001**	93 (1.9)	29 (0.7)	**<0.001**
Congestive Heart Failure	240484 (15.3)	218 (2.4)	**<0.001**	144 (2.9)	74 (1.9)	**0.004**
Periphral Vascular Disease	92633 (5.9)	110 (1.2)	**<0.001**	70 (1.4)	40 (1)	0.136
Cerebrovascular Disease	100292 (6.4)	177 (2)	**<0.001**	128 (2.6)	49 (1.3)	**<0.001**
Dementia	38832 (2.5)	64 (0.7)	**<0.001**	42 (0.8)	22 (0.6)	0.160
Connective Tissue Disease-Rheumatic Disease	18955 (1.2)	19 (0.2)	**<0.001**	13 (0.3)	6 (0.2)	0.397
Peptic Ulcer Disease	33875 (2.2)	44 (0.5)	**<0.001**	26 (0.5)	18 (0.5)	0.811
Mild Liver Disease	69022 (4.4)	1247 (14)	**<0.001**	414 (8.3)	833 (21.3)	**<0.001**
Diabetes without complications	282373 (18)	416 (4.7)	**<0.001**	249 (5)	167 (4.3)	0.133
Diabetes with complications	21259 (1.4)	22 (0.2)	**<0.001**	14 (0.3)	8 (0.2)	0.623
Paraplegia and Hemiplegia	14157 (0.9)	66 (0.7)	0.125	38 (0.8)	28 (0.7)	0.915
Renal Disease	106210 (6.8)	250 (2.8)	**<0.001**	149 (3)	101 (2.6)	0.296
Cancer	202342 (12.9)	597 (6.7)	**<0.001**	390 (7.8)	207 (5.3)	**<0.001**
Moderate or Severe Liver Disease	13067 (0.8)	296 (3.3)	**<0.001**	74 (1.5)	222 (5.7)	**<0.001**
Metastatic Carcinoma	62247 (4)	163 (1.8)	**<0.001**	107 (2.1)	56 (1.4)	**0.017**

Values were expressed as absolute number (percentage) and mean (95% confidence interval (95% CI). P-values were calculated by Chi-squared test and Mann-Whitney U test; and p-values in bold indicates statistically significant differences between groups. Statistically significant differences are shown in bold.Abbreviations: HCV, hepatitis C virus; HIV, human immunodeficiency virus.

In comparison with HIV-uninfected patients, HIV-infected patients were younger, less frequently male, had fewer comorbidities, had longer hospital admissions and were more frequently substance abusers especially tobacco and drugs (p<0.001) (Table 1A). In comparison with HIV/HCV-coinfected patients, HIV-monoinfected patients were slightly older, had longer hospital admissions and less frequently substance abusers (p<0.001) (Table 1B).

### Incidence of COPD (events per 10,000 person/year)

#### HIV-uninfected patients vs HIV-infected patients

**Fig 1:**
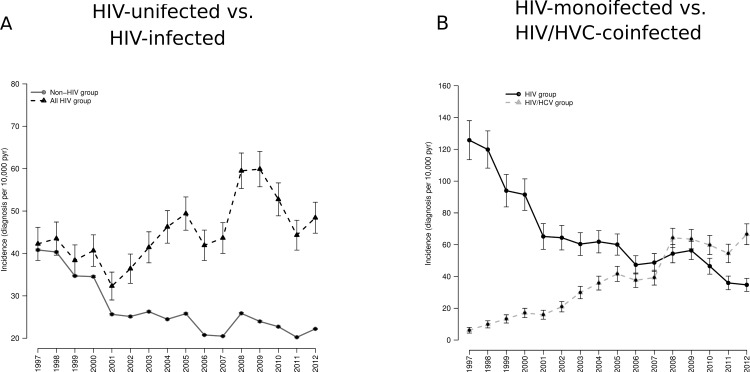
**Incidence of hospital admissions for COPD [HIV-infected patients vs. HIV-uninfected patients (A) and HIV-monoinfected patients vs. HIV/HCV-coinfected patients (B)] in Spain (1997–2012) stratified by HIV and HIV/HCV status.** Abbreviations: HCV, hepatitis C virus; HIV, human immunodeficiency virus.

The incidence rates of hospital admissions for COPD showed two different pattern in both groups along study period (Fig 1A and Table 2), being always higher among HIV-infected patients than in HIV-uninfected patients (45.4 vs.26.8; p<0.001). The incidence rate increased in HIV-infected patients during the study (41.4 (1997–1999) to 49.6 (2004–2012; p<0.001), while a dramatic decrease were observed in HIV-uninfected patients (38.6 (1997–199) to 22.9 (2004–2012); p<0.001) (Fig 1A and Table 2).

**Table 2:** Epidemiological trend of hospital admissions for COPD in Spain (1997 to 2012) stratified by calendar periods, HIV status and HIV/HCV status.

	Whole study period		1997–1999		2000–2003		2004–2012		Statistical significances		
	No.	Rate (95%CI)	No.	Rate (95%CI)	No.	Rate (95%CI)	No.	Rate (95%CI)	p ^(a)^	p ^(b)^	p ^(c)^
**COPD diagnosis (events per 10,000 person-yr)**											
**HIV-uninfected**	1571305	26.8 (26.8; 26.8)	389605	38.6 (38.5; 38.7)	390658	27.8 (27.8; 27.9)	791042	22.9 (22.9; 23)	**<0.001**	**<0.001**	**<0.001**
**HIV-infected**	8902	45.4 (44.5; 46.4)	1353	41.4 (39.1; 43.6)	1749	37.7 (36; 39.5)	5800	49.6 (48.3; 50.9)	**0.034**	**<0.001**	**<0.001**
**p-value** ^**(d)**^		**<0.001**		**0.012**		**<0.001**		**<0.001**			
**COPD diagnosis (events per 10,000 person-yr)**											
**HIV group**	5000	60.2 (58.6; 61.9)	1130	112.8 (106.2; 119.4)	1116	69.5 (65.4; 73.6)	2754	48.4 (46.6; 50.2)	**<0.001**	**<0.001**	**<0.001**
**HIV/HCV group**	3902	34.6 (33.5; 35.6)	223	9.8 (8.5; 11.1)	633	20.9 (19.3; 22.5)	3046	50.8 (49; 52.6)	**<0.001**	**<0.001**	**<0.001**
**p-value** ^**(d)**^		**<0.001**		**<0.001**		**<0.001**		0.057			

Values were expressed as absolute count; and rate (95% confidence interval (95% CI)). The p-values were calculated by the exact confidence intervals for incidence and mortality rates were calculated based on the Poisson distribution.

Significant differences:

(a), 1997–1999 vs. 2000–2003

(b), 1997–1999 vs. 2004–2010

(c), 2000–2003 vs. 2004–2010

(d), Differences between study groups

Statistically significant differences are shown in bold.

Abbreviations: HIV, human immunodeficiency virus.

#### HIV-monoinfected patient’s vs HIV/HCV-coinfected patients

On the other hand, the rates of COPD in HIV group and HIV/HCV group showed a different behavior (Fig 1B and Table 2). HIV/HCV group showed a dramatic increase in the incidence of hospital admissions for COPD during the study period (Fig 1B) been even 5 times higher in the last calendar period (9.8 (1997–1999) to 50.8 (2004–2012); p<0.001).

### In-hospital mortality among patients with COPD (deaths per 10,000 patients-yr)

#### HIV-uninfected patients vs HIV-infected patients

**Fig 2:**
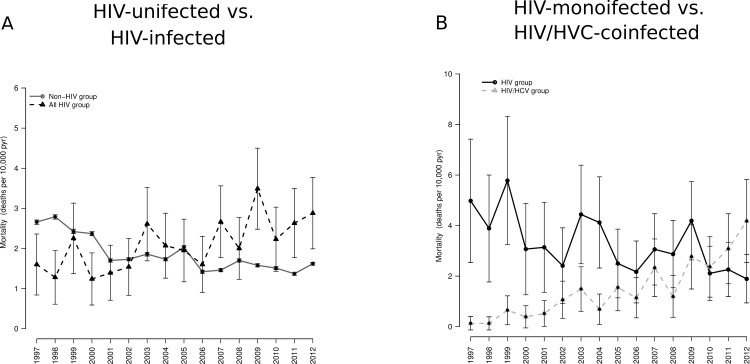
**Intrahospital mortality in patients admitted for COPD [HIV-infected patients vs. HIV-uninfected patients (A) and HIV-monoinfected patients vs. HIV/HCV-coinfected patients (B)] in Spain (1997–2012) stratified by HIV and HIV/HCV status.** Abbreviations: HCV, hepatitis C virus; HIV, human immunodeficiency virus.

The mortality was higher in HIV infected patients with COPD than in non HIV patients with COPD during the follow-up period (2.1 vs 1.8; p<0.001) (Fig 2A and Table 3). The mortality rate slightly decreased in non HIV patients with COPD during the study period (2.6 (1997–1999) to 1.6 (2004–2012); p<0.001), however, a moderate increase in mortality rate in all HIV infected patients with COPD was observed (1.7 (1997–1999) to 2.4 (2004–2012)).

**Table 3:** Epidemiological trend of intrahospital mortality of patients admitted for COPD in Spain (1997 to 2012) stratified by calendar periods, HIV status and HIV/HCV status.

	Whole study period		1997–1999		2000–2003		2004–2012		Statistical significances		
	No.	Rate (95%CI)	No.	Rate (95%CI)	No.	Rate (95%CI)	No.	Rate (95%CI)	p ^(a)^	p ^(b)^	p ^(c)^
**COPD mortality (events per 10,000 person-yr)**											
**HIV-uninfected**	108440	1.8 (1.8; 1.9)	26465	2.6 (2.6; 2.7)	26821	1.9 (1.9; 1.9)	55154	1.6 (1.6; 1.6)	**<0.001**	**<0.001**	**<0.001**
**HIV-infected**	416	2.1 (1.9; 2.3)	56	1.7 (1.3; 2.2)	79	1.7 (1.3; 2.1)	281	2.4 (2.1; 2.7)	0.999	0.050	**0.017**
**p-value** ^**(d)**^		**<0.001**		**<0.001**		0.306		**<0.001**			
**COPD mortality (events per 10,000 person-yr)**											
**HIV group**	258	3.1 (2.7; 3.5)	49	4.9 (3.5; 6.3)	53	3.3 (2.4; 4.2)	156	2.7 (2.3; 3.2)	0.146	**0.002**	0.731
**HIV/HCV group**	158	1.4 (1.2; 1.6)	7	0.3 (0.1; 0.5)	26	0.9 (0.5; 1.2)	125	2.1 (1.7; 2.5)	**0.032**	**<0.001**	**<0.001**
**p-value** ^**(d)**^		**<0.001**		**<0.001**		**<0.001**		**0.023**			

Values were expressed as absolute count; and rate (95% confidence interval (95% CI)). The p-values were calculated by the exact confidence intervals for incidence and mortality rates were calculated based on the Poisson distribution.

Significant differences:

(a), 1997–1999 vs. 2000–2003

(b), 1997–1999 vs. 2004–2010

(c), 2000–2003 vs. 2004–2010

(d), Differences between study groups

Statistically significant differences are shown in bold.

Abbreviations: HCV, hepatitis C virus; HIV, human immunodeficiency virus.

**Fig 3:**
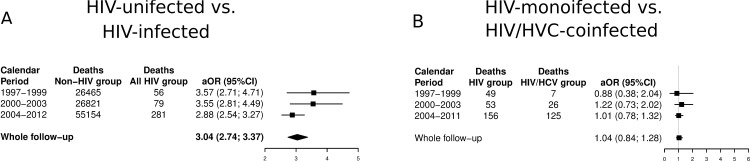
**Adjusted likelihood of death among patients admitted for COPD [HIV-infected patients vs. HIV-uninfected patients (A) and HIV-monoinfected patients vs. HIV/HCV-coinfected patients (B)] in Spain (1997–2012).** Abbreviations: HCV, hepatitis C virus; HIV, human immunodeficiency virus, aOR, adjusted odds ratio; 95%CI, 95% of confidence interval.

When adjusted logistic regression was performed, HIV patients with COPD had always higher likelihood of death than non HIV patients with COPD (aOR = 3.64; Fig 3A).

#### HIV-monoinfected patients vs HIV/HCV-coinfected patients

The mortality in the HIV group with COPD was higher than in HIV/HCV group with COPD during the follow-up period (3.1 vs. 1.4; p<0.001) (Fig 2B and Table 3). Nevertheless, a decrease in mortality rate were observed in the HIV group (4.9 (1997–1999) to 2.7 (2004–2012); p<0.001) while it increased in the HIV/HCV group more than 5 times from 1997–1999 period to 2004–2012 period (0.3 to 2.1; p<0.001) (Fig 2B and Table 3).

When adjusted logistic regression was performed no difference was observed in the likehood of death between the HIV and HIV/HCV groups (Fig 3B).

## Discussion

Our manuscript provided a nationwide view of COPD epidemiology in HIV-infected patients from the same healthcare system. The major findings were: 1) the HIV-infected patients had higher incidence rates of hospital admissions for COPD than the HIV-uninfected patients during the whole study period; 2) the HIV-monoinfected patients had higher incidence rates of hospital admissions for COPD than the HIV/HCV-coinfected patients in the early-period cART (1997–1999), but these rates decreased in the HIV-monoinfected patients and increased in the HIV/HCV-coinfected patients, being even similar in HIV/HCV-coinfected patients than in HIV-monoinfected patients in the late-period cART (2004–2011); 3) the HIV-infected patients had higher IHM than the HIV-uninfected patients; the likelihood of death was always at least 3 times higher in the HIV-infected patients than in the HIV-uninfected patients; 4) the IHM was higher in the HIV-monoinfected patients than in the HIV/HCV-coinfected patients in the early-period cART; however, in the late-period cART, the mortality rates trends seems higher in the HIV/HCV group. Furthermore, the likelihood of death for COPD in HIV/HCV-coinfected patients were similar than in HIV-monoinfected patients.

Few studies have assessed the association between HIV infection and diagnosis of COPD in the era of cART. One large study of- HIV-infected and HIV negative veterans found that HIV infection was an independent risk factor for COPD [35]. An analysis of a larger sample for the Veterans cohort extended these findings showing that the incidence of COPD was significantly higher in HIV-infected patients compared with those who were not HIV-infected. COPD was the most common non-infectious pulmonary disease in HIV-infected individuals, with a prevalence of 16% [3]. A similar figure was reported in another chart review of HIV-infected patients [36]. Although these studies reported a high prevalence of COPD diagnoses in HIV-infected individuals, these diagnoses were based on self-report or ICD-9 codes, as in our case, without measuring pulmonary function directly. Nevertheless, high COPD prevalence has been also reported among patients with HIV infection using spirometry to diagnose COPD, with figures from 6.8% to 21% [12,37–39]. In any case, COPD has been associated with increased risk of hospitalization among HIV infected patients [40].

It has been reported that HIV associated COPD occurs over a much shorter period of time than smoking related COPD. For example, emphysema may be recognized in 20–40 year old patients, rather in the 50–70 year old patients in non-HIV smokers [41]. In our study, we have demonstrated that, in comparison with HIV-uninfected patients, HIV-infected patients were younger. In addition, these patients were more frequently female, had fewer comorbidities, had longer hospital admissions and were more frequently substance abusers especially tobacco and drugs.

In a cohort of HIV-infected individuals, Kristoffersen et al reported that signs of obstructive lung disease were present in HIV-infected patients and seemed to increase over time [42]. HIV associated COPD may be more common in the cART era because it is frequently reported in patients without a history of HIV-related pulmonary complications and because the aging HIV-infected population has a longer exposure to smoking and HIV. In our study, we have also found that the incidence of COPD increased over time in HIV-infected patients, in contrast to trends reported in HIV-uninfected patients. Similar findings have been reported by other authors [16]. However, when we stratified to the HIV population according to HIV/HCV status, we observed that the incidence decreased over time in the HIV group, while it increased in HIV/HCV group, being similar in both groups the late-period cART (2004–2011).

A similar situation occurred with the death rate in our study. It was higher in the HIV-infected patients compared with those HIV-uninfected and increased significantly over time. However, when we stratified to the HIV population according to HIV/HCV status, we observed that the mortality decreased over time in the HIV group with COPD, while it increased in HIV/HCV group with COPD. It remains to assess the effect of eradication of HCV on pulmonary manifestations in HIV/HCV coinfected patients. In this sense, there is substantial evidence that successful antiviral therapy might reduce both hepatic and extrahepatic manifestations of HCV infection in HIV/HCV coinfected patients [43].

This study had several limitations that may have impacted our findings. First, this was a retrospective study and we had no access to patient clinical data (cART regimens, adherence, CD4+ cell counts, HIV viral load, COPD management, active hepatitis C), which could have helped us to more completely interpret our results. Second, due to the fact that MBDS data are anonymous, it is not possible to identify whether a patient has been hospitalized at different hospitals within the same calendar year. This may have caused a slight overestimation of our results, because we may have calculated disease recurrences as new participants. Besides, MBDS does not record the COPD-related deaths before admission to hospital. Third, we didn't used real figures of subjects infected with HIV in Spain from 1997 to 2011, because there was no national coverage data of HIV diagnoses in Spain in this period. Instead, we used an estimation provided by the National Centre of Epidemiology using the EPP software [26]. It was a similar process for the estimates of HCV coinfection from hepatitis C antibody status. Must be taken into mind that HCV antibodies become detectable at approximately 10 weeks post-infection, which would be within the acute HCV timeline. This could affect our estimated populations, but the data used for calculating estimated populations (hospital survey of HIV/AIDS infected patients and reports of two Spanish national cohorts (GeSIDA [31] and AMVACH [32]) are performed on patients with chronic HCV infection who came to the hospital. Moreover, by working with estimated populations, we were unable to calculate the incidence and mortality stratified by age and gender.

In conclusion, we found that in the cART era, COPD incidence and mortality have decreased among HIV-monoinfected individuals but have increased steadily among HIV/HCV-coinfected individuals.

## Abbreviations

cART: Combination antiretroviral therapy
HIV: Human immunodeficiency virus
CHC: Chronic hepatitis C
HCV: Hepatitis C virus
MBDS: Minimum Basic Data Set
ICD-9-CM: International Classification of Diseases, 9th ed, Clinical Modification
EPP: Estimation and Projection Package (EPP)
GeSIDA: “Grupo de Estudio de Sida”
AMVACH: Asociación Médica VACH de Estudios Multicentricos
95% CI: 95% confidence interval
aOR: Adjusted odds ratio
